# Preoperative prediction of microvascular invasion in hepatocellular carcinoma ≤5 cm based on contrast-enhanced ultrasound features and LI-RADS categorization: a multicenter study

**DOI:** 10.3389/fonc.2026.1901674

**Published:** 2026-07-16

**Authors:** Qian Zhang, Chuan Pang, Zhilong Liu, Ruining Wang, Wenwen Fan, Yufang Zhao, Yanjing Zhang, Ping Liang, Liping Liu

**Affiliations:** 1 Department of Interventional Ultrasound, First Hospital of Shanxi Medical University, Taiyuan, Shanxi, China; 2 Department of Medical Imaging, Shanxi Medical University, Taiyuan, Shanxi, China; 3 Department of Interventional Ultrasound, Fifth Medical Center of Chinese PLA General Hospital, Beijing, China

**Keywords:** hepatocellular carcinoma, LI-RADS, microvascular invasion, SonoVue, ultrasound

## Abstract

**Objectives:**

To investigate the predictive value of contrast-enhanced ultrasound (CEUS) features combined with Liver Imaging Reporting and Data System (LI-RADS) categorization for microvascular invasion (MVI) in hepatocellular carcinoma (HCC) ≤5 cm.

**Methods:**

This multicenter retrospective study enrolled adult patients with HCC ≤5 cm who underwent CEUS between January 2018 and December 2025. Least absolute shrinkage and selection operator (LASSO) regression and multivariate logistic analysis were employed to screen risk factors and establish the MVI prediction model. Three models were developed: a clinical model, an ultrasound model, and a combined model. The performance of the combined model was evaluated and validated using the area under the receiver operating characteristic (AUC), calibration curves, decision curve analysis (DCA), and the Hosmer-Lemeshow test.

**Results:**

A total of 261 patients with HCC ≤5 cm were enrolled. Patients were divided into a derivation cohort (n=209) and an external validation cohort (n=52). 85 patients (32.57%) were MVI-positive. LASSO regression and multivariate analysis revealed that AFP, tumor margin, enhanced homogeneity, mosaic, and LI-RADS were significantly associated with MVI. The combined model showed an AUC of 0.880 (95% CI: 0.832–0.929) in the derivation cohort and 0.832 (95% CI: 0.703–0.960) in the external validation cohort. Calibration curves revealed excellent agreement between the model’s predicted probability of MVI and the actual observed outcomes. DCA confirmed the excellent net benefits.

**Conclusion:**

This model can noninvasive preoperative prediction of MVI risk in patients with HCC ≤5 cm, offering a reliable reference for clinicians in developing personalized treatment strategies.

## 1 Introduction

According to data released by the International Agency for Research on Cancer, liver cancer accounts for over 750, 000 deaths annually, making it a leading cause of cancer-related mortality worldwide (1). Hepatocellular carcinoma (HCC), the most common type of primary liver cancer, has persistently high incidence rates (2). In recent years, rapid advancements in imaging have substantially improved the detection rate of liver cancer, but the prognosis remains unsatisfactory. It has been reported that the 5-year recurrence rate following curative liver resection ranges from 50% to 70%, while the 5-year recurrence rate for liver transplantation is approximately 15% to 20% (3–6).

During the development and progression of HCC, tumor cells exhibit a propensity for vascular invasion, leading to the formation of tumor thrombi. When major vessels are involved, tumor thrombi can be accurately diagnosed through imaging examinations. However, when microvessels are invaded, tumor thrombi are mostly located within the lumen of endothelial-lined vessels, which can only be detected microscopically (7, 8). This presents a significant challenge for noninvasive diagnosis. Studies have demonstrated that microvascular invasion (MVI) positive HCC is associated with higher aggressiveness and serves as a key risk factor for postoperative recurrence and unfavorable prognosis (8–10). Therefore, the ability to identify MVI noninvasively before surgery could provide clinicians with critical guidance in determining the extent of surgical resection and formulating personalized treatment strategies, which is of great significance for improving the prognosis of patients.

Contrast-enhanced ultrasound (CEUS), as a noninvasive, real-time and radiation-free imaging modality, can dynamically visualize the microvascular perfusion characteristics within the lesion and the surrounding liver parenchyma. It accurately reflects tumor vascularity patterns and hemodynamic alterations in the adjacent hepatic tissue, offering irreplaceable advantages in the preoperative assessment of HCC (3, 11). The Liver Imaging Reporting and Data System (LI-RADS), established by the American College of Radiology, provides a standardized imaging evaluation system for liver cancer (12). It enables consistent categorization of focal liver lesions, which closely correlates with the pathological features of HCC. LI-RADS has become an important basis for the clinical imaging diagnosis of liver cancer (13–15).

This study integrates CEUS features, LI-RADS, and clinical parameters to identify independent predictors of MVI, developing and validating a predictive model. The objective is to provide a more accurate and practical approach for the non-invasive preoperative assessment of MVI risk in patients with HCC ≤5 cm.

## 2 Materials and methods

### 2.1 Ethical statement

This retrospective study was approved by the Institutional Ethics Committee of the participating hospitals (No. KYLL 2023-132), and was compliant with both the Declarations of Helsinki and Istanbul. The requirement for informed consent was waived.

### 2.2 Participants

This multicenter, real-world retrospective study was conducted across 17 tertiary medicalinstitutions (**Supplementary Table 1**). Patients with HCC who underwent CEUS before surgery between January 2018 and December 2025 were enrolled.

Inclusion criteria: 1) age >18 years; 2) maximum tumor diameter ≤5.0 cm; 3) definitive pathological diagnosis confirmed by surgical resection; 4) no local or systemic anti-tumor therapy before CEUS; 5) preoperative examinations performed within 2 weeks before surgical intervention; 6) no allergies to any components of SonoVue.

Excluded criteria: 1) age ≤18 years; 2) maximum tumor diameter >5.0 cm; 3) patients with incomplete clinical data; 4) patients with inconclusive pathological diagnosis; 5) patients with missing or poor-quality ultrasound images.

**Figure 1:**
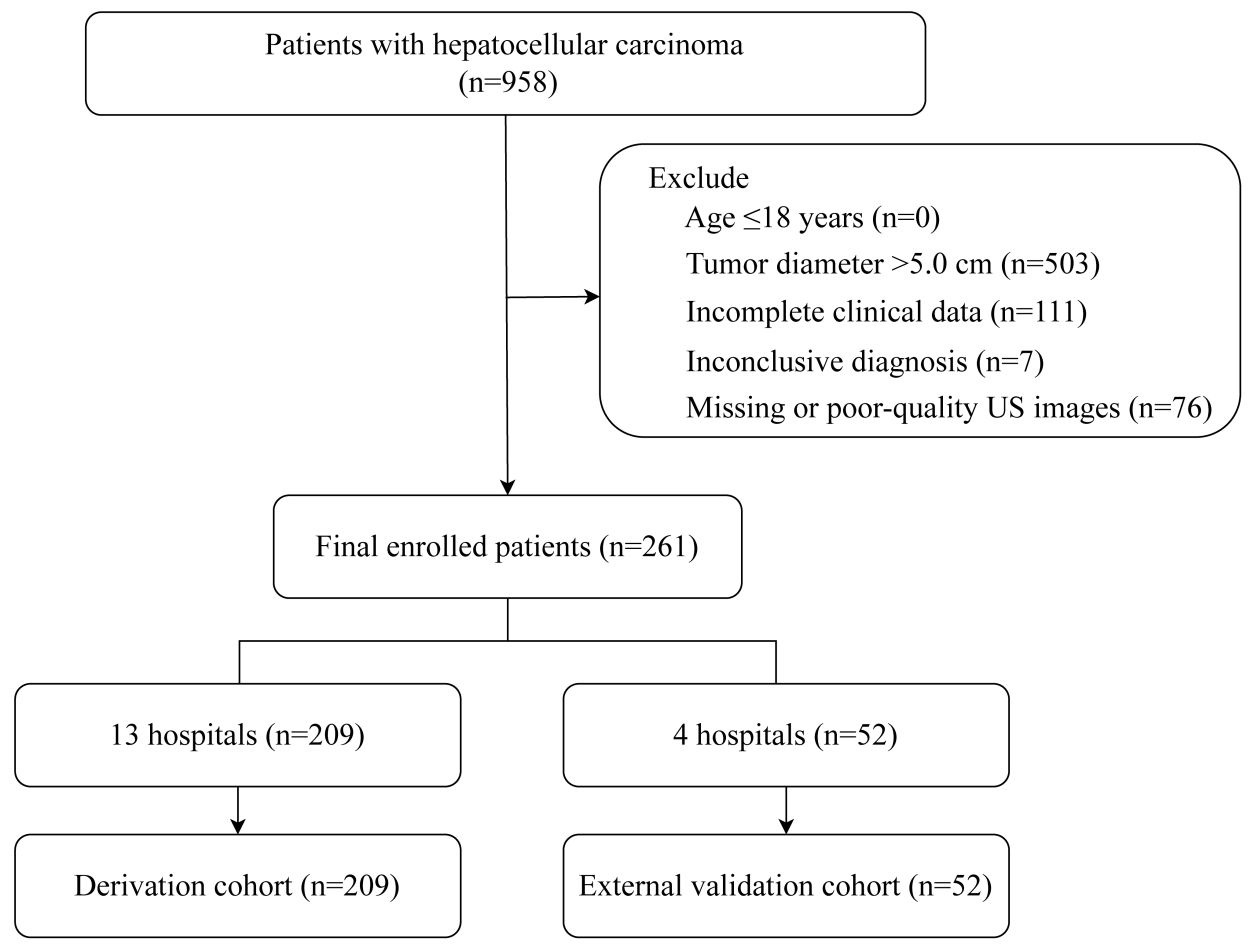
Research flowchart.

Patients were allocated to the derivation and external validation cohorts at a ratio of 4:1. To ensure the independence of the validation process, patients from the same institution were assigned exclusively to the same cohort. The flowchart is depicted in **Figure 1**.

### 2.3 Baseline clinical data

The baseline clinical data of the enrolled patients were collected, including age, gender, history of hepatitis, alpha-fetoprotein (AFP), white blood cells (WBC), platelets (PLT), alanine aminotransferase (ALT), and aspartate aminotransferase (AST).

MVI refers to the presence of clusters of cancer cells in the vascular lumen with endothelial cell linings under the microscope (7, 16).

### 2.4 Ultrasound and CEUS feature

Ultrasonography was performed using a convex broadband probe (frequency range, 3.5--5.0 MHz). The probe was positioned to clearly visualize the target lesion and the surrounding liver parenchyma. After stabilizing the probe position, switch to CEUS mode with a low mechanical index. A bolus of 1.0--2.4 ml of SonoVue (Bracco, Milan, Italy) was injected via the elbow vein, immediately followed by 5 ml of saline. The entire examination was strictly timed, with continuous recording for 2 minutes post-injection. Subsequently, dynamic images were stored at 1-minute intervals until 6 minutes. All imaging data were saved in DICOM format.

Ultrasound features were gleaned from records, including 1) tumor size, defined as the maximum diameter; 2) location; 3) tumor shape; 4) tumor margin; 5) tumor echogenicity; 6) blood flow signal; 7) portal hypertension; 8) splenomegaly; 9) ascites.

CEUS features were also collected and analyzed: 1) enhanced homogeneity, defined as the uniformity of microbubble perfusion zones during the peak enhancement phase excluding non-enhanced areas within the tumor), categorized as homogeneous and heterogeneous; 2) capsular enhancement; 3) mosaic architecture, characterized by the random distribution of internal nodules or compartments, typically accompanied by varying imaging features; 4) enhanced margin; 5) enhanced morphology; 6) necrotic, defined as the presence of non-enhanced areas within the tumor; 7) LI-RADS category, assessed and classified according to the CEUS LI-RADS v2017 (12).

All data were independently assessed by 2 researchers with 5 years of experience in abdominal radiology. Discrepancies were resolved by consensus with a third radiologist with 25 years of experience.

### 2.5 Sample size

In the development of multivariate predictive models, sample size is conventionally determined with reference to the events per variable (EPV), which is defined as the ratio of the number of outcome events to the number of candidate predictor variables. An empirical study recommended a minimum EPV of 10 to mitigate the risk of overfitting (17). This criterion has since been widely adopted in clinical prediction modeling. Applying this standard, we constrained a maximum of candidate predictors to 6 variables (69 outcome events/10 EPV).

### 2.6 Statistical analyses

All statistical tests were performed and plotted using *Python* (version 3.10), *R* (version 4.2.2) and *Free Statistics* (version 2.4). A two-sided test *P* value of less than 0.05 was considered significant.

To ensure the robustness of the analysis and minimize potential bias, patients with incompletedata were excluded from the final dataset. Continuous variables with normal distribution were presented as means ± SD and analyzed by t-test. Non-normally distributed continuous variables were expressed as median and inter-quartile range and analyzed using the Mann-Whitney U test. Categorical factors were described as frequencies or proportions, and analyzed by χ^2^ test or Fisher’s exact test. Inter-observer agreement was assessed using the kappa (κ) statistic, as detailed in the **Supplementary Table 2**. Agreement was classified as as poor (0-0.20), fair (0.21-0.40), moderate (0.41-0.60), good (0.61-0.80), or excellent (0.81-1.00).

The least absolute shrinkage and selection operator (LASSO) regression was used in the derivation cohort to identify potential predictors for MVI. The optimal value of λ was determined via 10-fold cross-validation, with the λ.1se criterion applied to select the final features. These features were subsequently used in univariate and multivariate logistic regression analyses to identify statistically significant predictors, which were then used to construct a nomogram. We developed 3 models: a clinical model (C model), an ultrasound model (U model), and a combined model (Com model).

We used the area under the receiver operating characteristic (AUC) for assessing the differentiation of the models, and calculated accuracy, sensitivity, specificity, precision and the Youden index. Internal validation was performed with the bootstrap resampling method (500 times). Calibration was assessed using the Hosmer-Lemeshow test and visualized with calibration curves. The decision curve analysis (DCA) was used to evaluate the net clinical benefits. In the external validation cohort, the model was tested to assess its generalizability to various populations. Model performance was evaluated using ROC, calibration curves, and DCA. Finally, the Brier score was calculated to assess the overall accuracy of the prediction model. Pairwise comparisons of model performance were conducted statistically using the DeLong test.

## 3 Results

### 3.1 Participants characteristics

The study design flowchart is illustrated in **Figure 1**. A total of 958 patients with HCC were initially enrolled. After applying inclusion and exclusion criteria, 261 patients were ultimately included (55.35 ± 10.32 years, 213 males). The derivation cohort consisted of 209 patients (54.38 ± 10.32 years, 174 males), while the external validation cohort comprised 52 patients (59.23 ± 9.45 years, 39 males) from an independent dataset. The baseline characteristics of patients in both cohorts are described in **Table 1**. In the two cohorts, there were 69 (33.01%) and 16 (30.77%) patients confirmed as MVI-positive respectively, with no statistically significant difference (*P*>0.05). No significant differences were observed between the two cohorts in any other characteristics except age (all *P*>0.05). To evaluate whether age modified the model’s performance, we tested the interaction between age and the predicted risk score in both cohorts; the interaction *P*-values were 0.832 and 0.566, respectively.

**Table 1:** Baseline characteristics.

Variables	Derivation cohort (n=209)	External validation cohort (n=52)	*P*
Age	54.38 ± 10.32	59.23 ± 9.45	0.002
Gender			0.169
Female	35 (16.75)	13 (25.00)	
Male	174 (83.25)	39 (75.00)	
History of hepatitis			0.066
Presence	151 (72.25)	44 (84.62)	
Absence	58 (27.75)	8 (15.38)	
AFP, ng/mL			0.787
≤400	157 (75.12)	40 (76.92)	
>400	52 (24.88)	12 (23.08)	
WBC, /L			0.418
≤4*10⁹	49 (23.44)	15 (28.85)	
>4*10⁹	160 (76.56)	37 (71.15)	
PLT, /L			0.908
≤100*10⁹	62 (29.67)	15 (28.85)	
>100*10⁹	147 (70.33)	37 (71.15)	
ALT, U/L			0.958
≤44	156 (74.64)	39 (75.00)	
>44	53 (25.36)	13 (25.00)	
AST, U/L			0.898
≤44	159 (76.08)	40 (76.92)	
>44	50 (23.92)	12 (23.08)	
Tumor size	3.08 ± 1.02	3.08 ± 1.06	0.973
Location			0.847
Left lobe	55 (26.32)	13 (25.00)	
Right lobe	154 (73.68)	39 (75.00)	
Tumor shape			0.291
Regular	163 (77.99)	44 (84.62)	
Irregular	46 (22.01)	8 (15.38)	
Tumor margin			0.549
Clear	148 (70.81)	39 (75.00)	
Unclear	61 (29.19)	13 (25.00)	
Tumor echogenicity			0.099
Hypo	106 (50.72)	33 (63.46)	
Iso/Hyper/Mixed	103 (49.28)	19 (36.54)	
Blood flow signal			0.438
Negative	104 (49.76)	29 (55.77)	
Positive	105 (50.24)	23 (44.23)	
Portal hypertension			0.088
Negative	173 (82.78)	48 (92.31)	
Positive	36 (17.22)	4 (7.69)	
Splenomegaly			0.078
Negative	157 (75.12)	45 (86.54)	
Positive	52 (24.88)	7 (13.46)	
Ascites			0.462
Negative	201 (96.17)	49 (94.23)	
Positive	8 (3.83)	3 (5.77)	
Enhanced homogeneity			0.662
Homogeneous	163 (77.99)	42 (80.77)	
Heterogeneous	46 (22.01)	10 (19.23)	
Capsular enhancement			0.435
Negative	141 (67.46)	38 (73.08)	
Positive	68 (32.54)	14 (26.92)	
Mosaic			0.547
Negative	187 (89.47)	45 (86.54)	
Positive	22 (10.53)	7 (13.46)	
Enhanced margin			0.770
Well defined	149 (71.29)	36 (69.23)	
Poorly defined	60 (28.71)	16 (30.77)	
Enhanced shape			0.963
Regular	144 (68.90)	36 (69.23)	
Irregular	65 (31.10)	16 (30.77)	
Necrotic			0.069
Negative	161 (77.03)	46 (88.46)	
Positive	48 (22.97)	6 (11.54)	
LI-RADS			0.983
M	68 (32.54)	17 (32.69)	
4/5	141 (67.46)	35 (67.31)	
MVI			0.757
Positive	69 (33.01)	16 (30.77)	
Negative	140 (66.99)	36 (69.23)	

### 3.2 Model development

**Figure 2:**
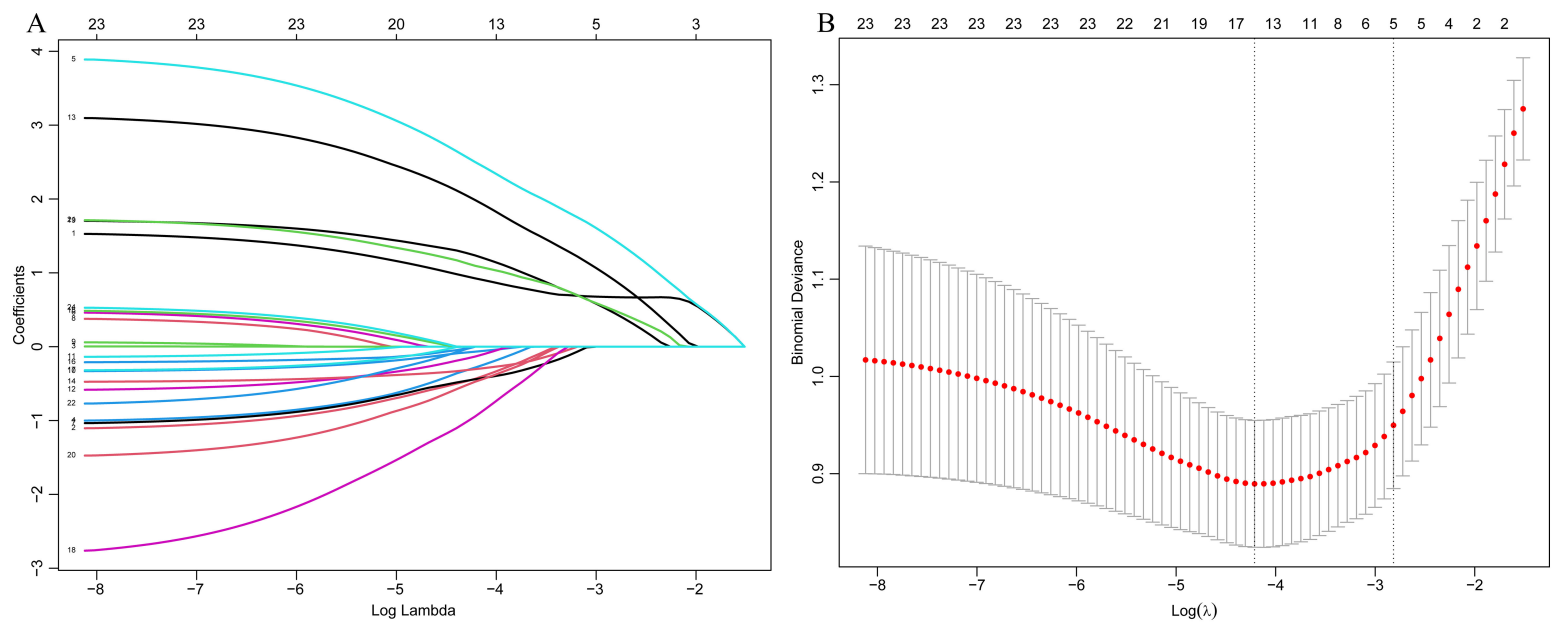
Features selection using LASSO. LASSO regression plot. **(A)** LASSO coefficient profiles. Each curve represents the LASSO coefficient profile of a feature across the log (λ) sequence. **(B)** Partial likelihood deviance plot for optimal parameter (λ) selection.

LASSO regression analysis was employed to identify potential predictors of MVI, as shown in **Figure 2**. The selected features were then incorporated into multivariate logistic regression analysis. The results indicated that AFP, tumor margin, enhanced homogeneity, mosaic and LI-RADS were independent risk factors for MVI in patients with HCC ≤5 cm, as summarized in **Table 2**.

**Table 2:** Logistic univariate and multivariate proportional hazard models of risk factors.

Variable	Univariate		Multivariate	
	OR (95CI%)	*P*	OR (95CI%)	*P*
AFP (>400 ng/mL)	9.635 (4.715~19.690)	<0.001	23.030 (7.497~70.740)	<0.001
Tumor margin (Unclear)	3.667 (1.956~6.872)	<0.001	13.247 (4.803~36.540)	<0.001
Enhanced homogeneity (Heterogeneous)	3.628 (1.840~7.155)	<0.001	5.269 (1.841~15.078)	0.002
Mosaic (Positive)	8.827 (3.098~25.154)	<0.001	4.547 (1.037~19.931)	0.045
LI-RADS (M)	8.507 (4.401~16.441)	<0.001	2.698 (1.152~6.316)	0.022

**Figure 3:**
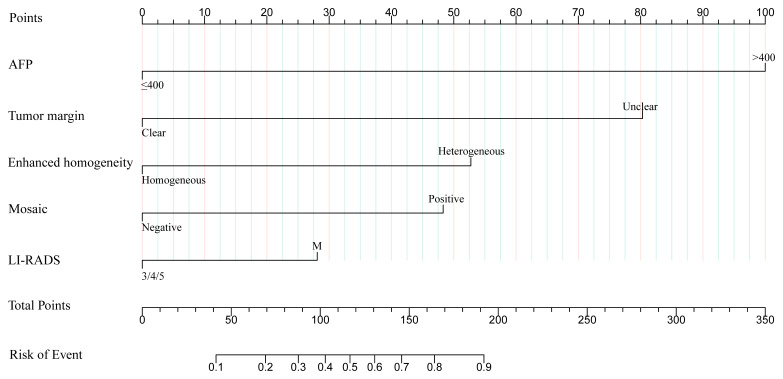
Nomogram for predicating MVI in HCC ≤5cm.

**Figure 4:**
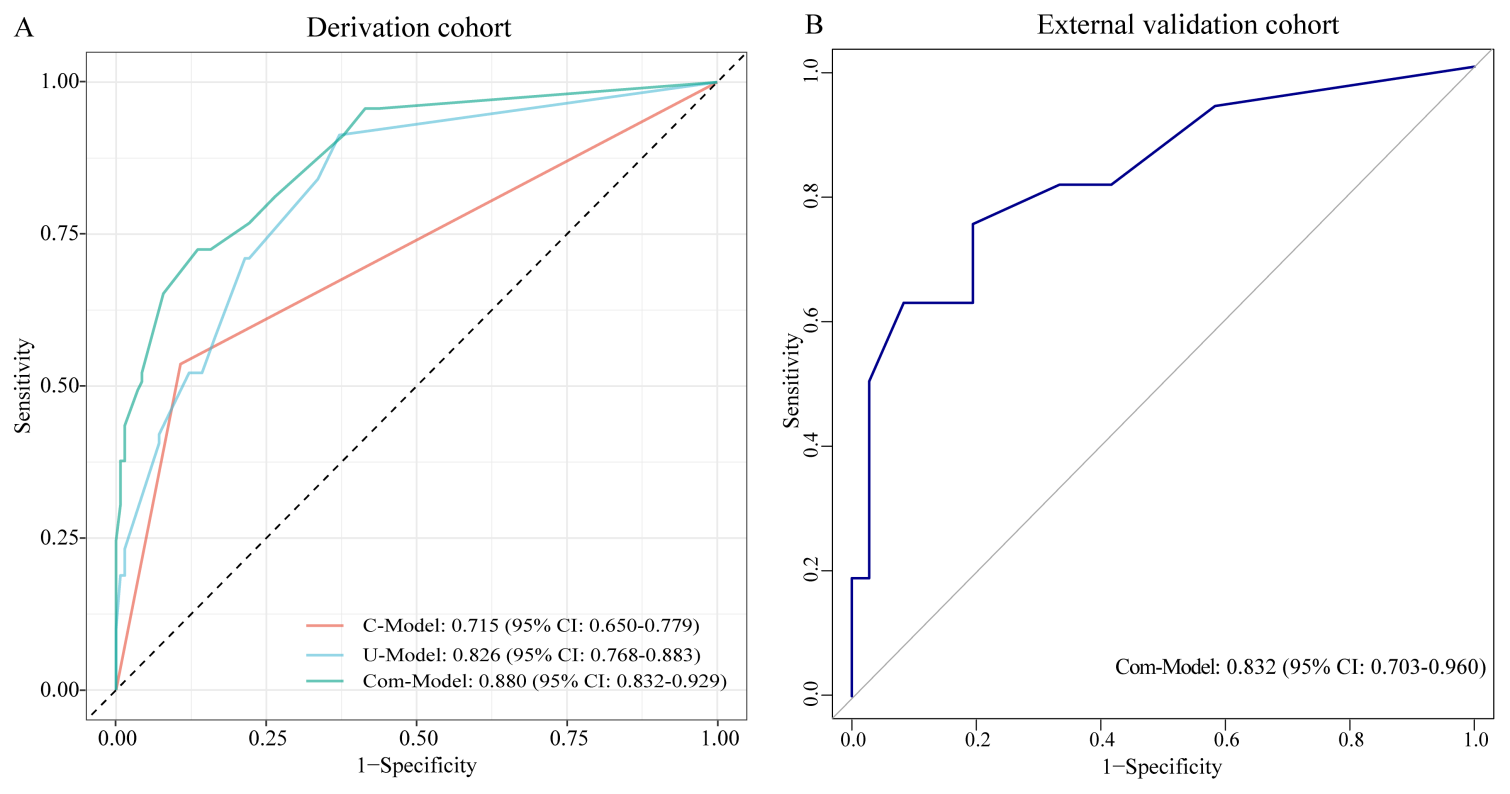
ROC curves for the prediction models in the derivation and external validation cohorts. **(A)** In the derivation cohort, ROC curve for the C model, U model and Com model. **(B)** In the external validation cohort, ROC curve for the Com model.

In the derivation cohort, the C model contained 1 clinical predictor: AFP. Its AUC for predicting MVI in HCC ≤5 cm was 0.715 (95% CI: 0.650-0.779), with an accuracy of 0.775, sensitivity of 0.536, specificity of 0.893 and precision of 0.712. The U model was constructed by integrating 4 ultrasound and CEUS predictors, including tumor margin, enhanced homogeneity, mosaic and LI-RADS. The model demonstrated an AUC of 0.826 (95% CI: 0.768-0.883), with accuracy, sensitivity, specificity and precision of 0.723, 0.913, 0.629 and 0.548, respectively. The Com model included all 5 independent predictors and was presented as a nomogram (**Figure 3**). The model achieved an AUC of 0.880 (95% CI: 0.832-0.929), with accuracy, sensitivity, specificity and precision of 0.818, 0.725, 0.864 and 0.725, respectively. The Com model demonstrated superior predictive performance compared to the other models, as illustrated in **Figure 4A**; **Table 3**. Interobserver agreement demonstrated excellent reliability between observers in the **Supplementary Table 2**. **Supplementary Table 3** presented the results of pairwise statistical comparisons of the AUCs among different models using the DeLong test.

**Table 3:** Comparison of the predictive ability of 3 models in the derivation cohort.

Model	AUC	95% CI	Accuracy	Sensitivity	Specificity	Precision	Youden
C-Model	0.715	0.650-0.779	0.775	0.536	0.893	0.712	0.429
US-Model	0.826	0.768-0.883	0.723	0.913	0.629	0.548	0.542
Com-Model	0.880	0.832-0.929	0.818	0.725	0.864	0.725	0.589

### 3.3 Model evaluation and validation

**Figure 5:**
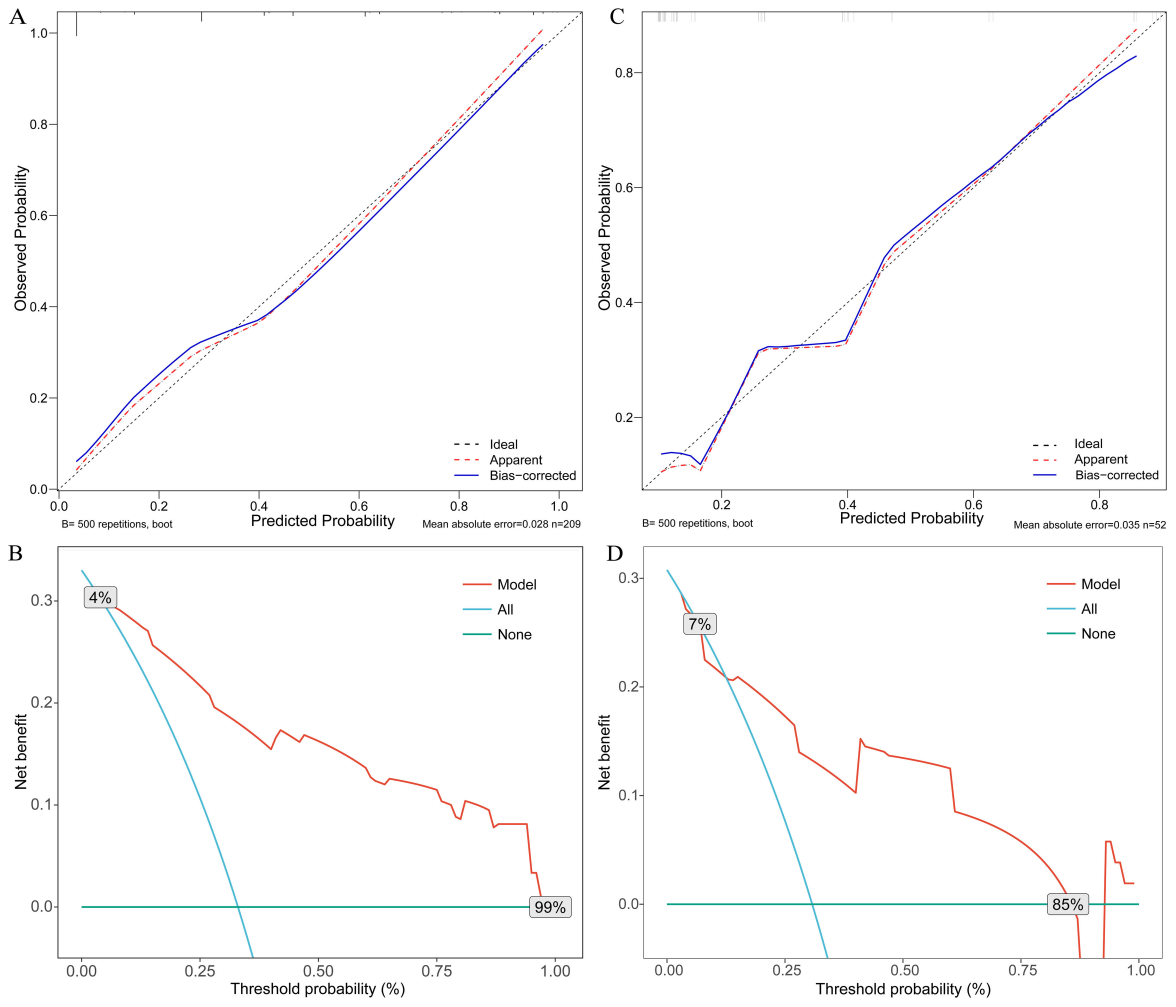
Performance evaluation of the Com model. **(A)** Calibration curve in the derivation cohort; **(B)** Decision curve analysis in the derivation cohort; **(C)** Calibration curve in the external validation cohort; **(D)** Decision curve analysis in the external validation cohort.

We conducted internal and external validation to assess the Com model’s discriminatory ability. Internal validation was performed using 500 bootstrap resampling, with an AUC of 0.872. Calibration curve demonstrated excellent agreement between predicted probabilities and observed outcomes (**Figure 5A**). DCA evaluated the clinical value of the prediction model, indicating that with 4%-99% probabilities, its application provided a greater net benefit compared to the treat-all or treat-none strategies (**Figure 5B**). Hosmer-Lemeshow goodness-of-fit test indicated excellent fit (*P* = 0.817). The Brier score was 0.134, suggesting acceptable model calibration.

In the external validation cohort, the Com model achieved an AUC of 0.832 (95% CI: 0.703-0.960) for predicting MVI in HCC ≤5 cm (**Figure 4B**). Calibration curves showed good concordance between observed and predicted risks (**Figure 5C**). DCA demonstrated that the model provided a greater net benefit compared to treat-all or treat-none strategies within a 7%–85% threshold probability range (**Figure 5D**). The Hosmer-Lemesow test confirmed good fit (*P* = 0.489). And the Brier score of 0.146 indicated that the model was well calibrated.

## 4 Discussion

For patients with early-stage HCC, curative treatments such as surgical resection or liver transplantation are generally more favorable. However, in the subgroup of patients with HCC ≤5 cm, the presence or absence of MVI leads to substantial prognostic differences, with MVI-positive patients experiencing worse prognoses (18). Therefore, our study aims to accurately predict the risk of MVI preoperatively in patients with HCC ≤5 cm, thereby providing a reliable reference for developing personalized treatment strategies in clinical practice.

In this multicenter study, we developed and validated a predictive model based on CEUS features and LI-RADS categorization to accurately estimate the risk of MVI in patients with HCC ≤5 cm. The identified predictors included AFP, tumor margin, enhanced homogeneity, mosaic architecture and LI-RADS. The model achieved an AUC of 0.880 (95% CI: 0.832-0.929) in the derivation cohort and 0.832 (95% CI: 0.703–0.960) in the external validation cohort. Bootstrap validation supported the reliability of the model, with an adjusted AUC of 0.872. Calibration curves demonstrated excellent agreement between the predicted probabilities and observed outcomes, and the Hosmer-Lemeshow test showed *P* = 0.817 and 0.489. Decision curve analysis further confirmed favorable net clinical benefit.

AFP is an important serum biomarker for the screening, diagnosis, and prognostic evaluation of HCC. Its levels are closely correlated with tumor malignancy and invasiveness (19, 20). AFP exerts dual oncogenic effects by promoting tumor cell proliferation and inhibiting HCC cell apoptosis (21). High AFP expression may enhance the invasive capacity of tumor cells by inducing epithelial-mesenchymal transition (EMT), thereby promoting MVI (22). In this study, AFP >400 ng/mL was identified as an independent risk factor for MVI in patients with HCC ≤5 cm, which is consistent with previous findings that elevated AFP levels indicate increased tumor aggressiveness (23). Furthermore, our study confirmed that unclear tumor margin was an independent predictor of MVI. HCC is often surrounded by a pseudocapsule, which is primarily composed of dense fibrous tissue and compressed adjacent hepatic parenchyma. When MVI occurs, tumor cells invade and disrupt the pseudocapsule, leading to its incompleteness or absence, which consequently results in unclear tumor margin. Similar findings have also been reported by Hwang YJ et al. (24).

CEUS is one of the imaging methods recommended by the European Federation of Societies for Ultrasound in Medicine and Biology (EFSUMB) guidelines and the Chinese guidelines for the diagnosis and treatment of HCC for the routine preoperative evaluation of focal liver lesions (3, 25). CEUS accurately reflects the microvascular perfusion characteristics of tumors, offering irreplaceable advantages in the preoperative diagnosis of HCC. In MVI-positive patients, CEUS demonstrates heterogeneous enhancement. This finding is primarily attributed to tumor cells invasion into surrounding microvessels, leading to luminal obstruction or the formation of disorganized neovessels. Luminal obstruction by tumor emboli often leads to necrosis. Meanwhile, the newly formed vessels exhibit chaotic architecture and incomplete walls, causing disordered contrast agent perfusion, ultimately manifesting as heterogeneous enhancement.

Mosaic architecture reflects tumor heterogeneity resulting from variations in tissue differentiation, molecular characteristics, and patterns of tumor infiltration and growth (26). LI-RADS M category was originally designed to encompass a range of non-HCC malignancies. However, in clinical practice, atypical HCCs are also classified as LI-RADS M (27). These tumors exhibit distinctive imaging features, including rim arterial phase hyperenhancement (APHE), early washout, or marked washout. Rim APHE suggests the presence of active, structurally disordered neovascularization at the tumor periphery, which is often associated with aggressive biological behavior. Early or marked washout reflects the existence of abundant arteriovenous shunts or abnormal draining vessels within the tumor. Such vascular structures exhibit hemodynamic instability, and their fragile walls are more susceptible to disruption by cancer cells, thereby creating conditions conducive to MVI. This patho−hemodynamic link provides a biologically plausible explanation for the strong association between LI−RADS M categorization and MVI observed. A study by Huang JY et al. also confirmed that the earlier the tumor washout, the higher the risk of MVI (28). Previous studies have showed that LI-RADS M is an independent risk factor for HCC, typically correlating with poorly differentiated and highly aggressive tumor (29). In this study, LI-RADS classification was identified as an independent predictor of MVI in patients with HCC ≤5 cm, which was consistent with the findings of Fei X et al. (30). Nevertheless, while CEUS LI-RADS demonstrates high diagnostic performance for HCC, its utility in predicting MVI remains to be further investigated.

It should be acknowledged that the external validation cohort in this study had a significantly older age distribution than the training cohort. The age difference between the two cohorts primarily stems from the inherent heterogeneity of populations across the multicenter institutions. In fact, such inter-population variation more closely mirrors the real-world diversity encountered in routine clinical practice, thus provides an opportunity to assess the model’s generalizability across different demographic settings. It should be noted that age was not selected as an independent predictor in our final model, suggesting that its direct contribution to the predicted outcome may be limited. To evaluate its potential confounding effect, we tested the interaction between age and the predicted risk score in both cohorts. The interaction *P*-values were 0.832 and 0.566, respectively, indicating that age did not significantly modify the model’s predictive accuracy. Nevertheless, we acknowledge that residual confounding cannot be entirely excluded, and future validation in more age−balanced cohorts would help to further substantiate our findings.

There are several limitations in this study. First, as a retrospective multicenter study, inter-institutional differences are unavoidable. To minimize measurement variability, standardized imaging protocols were used at all institutions. The robustness of the proposed model warrants further validation through large−scale, multicenter prospective studies. In addition, the external validation cohort was of a relatively modest size. Further validation in larger, independent cohorts will be essential to definitively ascertain the model’s performance. What’s more, the parameters employed in this study relied on physician interpretation, which may introduce a degree of subjectivity. In the future, we will focus on integrating ultrasound radiomic features to further enhance objectivity and reproducibility.

## 5 Conclusion

In conclusion, we developed and validated a reliable and user-friendly clinical model that integrates CEUS and LI-RADS features to accurately and noninvasively predict the risk of MVI in patients with HCC ≤5 cm. It could assist clinicians in objectively selecting optimal treatment strategies, thereby improving clinical outcomes.

## Data Availability

The raw data supporting the conclusions of this article will be made available by the authors, without undue reservation.
