# Supplementary material for: Preoperative prediction of microvascular invasion in hepatocellular carcinoma ≤5 cm based on contrast-enhanced ultrasound features and LI-RADS categorization: a multicenter study
Source: Front Oncol. 2026 Jul 16;16:1901674. doi: 10.3389/fonc.2026.1901674 (PMC13422560; doi:10.3389/fonc.2026.1901674)
Supplement: Supplementary file 1 [file Table1.docx]

**Supplementary Table 1. The number of patients enrolled in each cohort.**

| **Institution name** | **Numbers** |
| --- | --- |
| **Derivation cohort** |  |
| The Third Affiliated Hospital of Sun Yat-sen University | 42 |
| Xiangya Hospital, Central South University | 29 |
| Fujian Provincial Cancer Hospital | 24 |
| The Affiliated Hospital of Qingdao University | 21 |
| The First Affiliated Hospital of Zhejiang University | 17 |
| The Second Affiliated Hospital of Kunming Medical University | 16 |
| Hunan Provincial People's Hospital | 13 |
| Xijing Hospital, The Fourth Military Medical University | 11 |
| Tangdu Hospital, The Fourth Military Medical University | 9 |
| The Third Central Hospital of Tianjin | 8 |
| The Second Affiliated Hospital of Harbin Medical University | 7 |
| Qinghai University Affiliated Hospital | 7 |
| Jining First People's Hospital | 5 |
| **External validation cohort** |  |
| The First Hospital of Shanxi Medical University | 27 |
| Sun Yat-sen Memorial Hospital | 15 |
| Jilin University Sino Japanese Friendship Hospital | 6 |
| Shanxi Provincial Cancer Hospital | 4 |
