# Supplementary material for: Preoperative prediction of microvascular invasion in hepatocellular carcinoma ≤5 cm based on contrast-enhanced ultrasound features and LI-RADS categorization: a multicenter study
Source: Front Oncol. 2026 Jul 16;16:1901674. doi: 10.3389/fonc.2026.1901674 (PMC13422560; doi:10.3389/fonc.2026.1901674)
Supplement: Supplementary file 2 [file Table2.docx]

**Supplementary Table 2. Inter-observer agreement analysis of image features.**

| Variables | κ | *P* |
| --- | --- | --- |
| Enhanced homogeneity | 0.872 | <0.001 |
| Capsular enhancement | 0.789 | <0.001 |
| Mosaic | 0.866 | <0.001 |
| Enhanced margin | 0.869 | <0.001 |
| Enhanced shape | 0.864 | <0.001 |
| Necrotic | 0.918 | <0.001 |
| LI-RADS | 0.891 | <0.001 |
