# Supplementary material for: Preoperative prediction of microvascular invasion in hepatocellular carcinoma ≤5 cm based on contrast-enhanced ultrasound features and LI-RADS categorization: a multicenter study
Source: Front Oncol. 2026 Jul 16;16:1901674. doi: 10.3389/fonc.2026.1901674 (PMC13422560; doi:10.3389/fonc.2026.1901674)
Supplement: Supplementary file 3 [file Table3.docx]

**Supplementary Table 3. Comparison of model AUCs using the DeLong test.**

| Model 1 | Model 2 | AUC 1 | AUC 2 | *P* |
| --- | --- | --- | --- | --- |
| C-Model | US-Model | 0.715 | 0.826 | 0.009 |
| C-Model | Com-Model | 0.715 | 0.880 | <0.001 |
| US-Model | Com-Model | 0.826 | 0.880 | 0.006 |
